# CNN-LSTM Facial Expression Recognition Method Fused with Two-Layer Attention Mechanism

**DOI:** 10.1155/2022/7450637

**Published:** 2022-10-13

**Authors:** Ye Ming, Hu Qian, Liu Guangyuan

**Affiliations:** ^1^College of Artificial Intelligence, Southwest University, Chongqing, China; ^2^College of Electronic Information Engineering Southwest University, Chongqing, China

## Abstract

When exploring facial expression recognition methods, it is found that existing algorithms make insufficient use of information about the key parts that express emotion. For this problem, on the basis of a convolutional neural network and long short-term memory (CNN-LSTM), we propose a facial expression recognition method that incorporates an attention mechanism (CNN-ALSTM). Compared with the general CNN-LSTM algorithm, it can mine the information of important regions more effectively. Furthermore, a CNN-LSTM facial expression recognition method incorporating a two-layer attention mechanism (ACNN-ALSTM) is proposed. We conducted comparative experiments on Fer2013 and processed CK  + datasets with CNN-ALSTM, ACNN-ALSTM, patch based ACNN (pACNN), Facial expression recognition with attention net (FERAtt), and other networks. The results show that the proposed ACNN-ALSTM hybrid neural network model is superior to related work in expression recognition.

## 1. Introduction

In the field of facial expression recognition based on deep learning networks, the convolutional neural network (CNN) has been proven to be an important means of extracting local spatial features of images. However, CNN is a typical feedforward deep network. Its network structure is monotonously connected and the information flows in only one direction, which makes it lack the recognition of contextual timing information and restricts the accuracy of the algorithm.

The recurrent neural network (RNN) has loops because neurons in them not only receive information from other neurons but also receive information from themselves. The input of RNN is sequence data, and a loop unit is executed at each time t. The calculation of the hidden layer at the next time t+1 depends on the current input data and the hidden state at time t [1]. Since each hidden state saves the node information that is before time t, RNN can establish the global dependency of sequence data. RNN is used in tasks such as speech recognition and language generation.

Computer vision problems involving sequence input can be satisfying solved by RNN. As for multilabel image classification tasks [2], Zhang et al. found that the traditional multilabel image classification method cannot explicitly use the label dependency in the image. It can be seen that on the basis of RNN, CNN-RNN shows a relationship between semantics and image tags. Guo et al. used CNN to gain the discriminative features and used RNN to optimize the classification of coarse and fine labels [3]. Then, the classification performance of traditional hierarchical models is improved by fusing hierarchical information.

Parameters of RNN are adjusted through the back propagation algorithm. However, the network likely experience gradient disappearance as the length of the sequence data input to the network increases. Long short-term memory network (LSTM) introduces gating units to control the accumulation speed of information [1]. This not only endows it with a stronger memory capacity for storing sequence information but also effectively deals with the disappearance of gradients, which is an improvement to RNN. LSTM has been widely used in image annotation, machine translation, semantic recognition, and other directions [4].

When using neurons as information storage units in neural networks, the more information is stored, the more neurons are needed and the corresponding networks structure are more complex [5]. A large amount of information in the networks often leads to information overload, so the attention mechanism is introduced for this problem. The attention mechanism draws on the basic principles of human brain resource allocation [6]. To obtain richer information from limited resources, more attention is paid to important areas for human attention is limited. Initially, the attention mechanism was proposed in the field of computer vision and later also used in the field of natural language processing [7–9]. Its main purpose is to rationally use computing resources. The method is to allocate more attention to the key parts. This effectively solves the problem of information redundancy and prevents the loss of key information.

Based on the ability of the attention mechanism to focus on key parts and the excellent memory capability of the cyclic neural network, this paper proposes a CNN-LSTM hybrid neural network that integrates a two-layer attention mechanism (ACNN-ALSTM) [10, 11]. The CNN-LSTM network abstracts the local feature information from the facial expression image and establishes the global spatial dependence of the features through the LSTM. At the same time, the two-layer attention mechanism is introduced to mine the information of essential areas to obtain more discriminative expression features, thereby improving the recognition accuracy.

Our contributions are summarized as follows:

1. A two-layer attention mechanism is introduced to propose a novel ACNN-ALSTM model for facial expression recognition. The two-layer attention mechanism can enhance attention to key areas.
2. The LSTM algorithm is introduced to establish the global spatial dependency of facial information. Afterwards, the effect of different network layers and the number of nodes in the hidden layer of the LSTM model on expression recognition are investigated.
3. Comparative experiments are made to verify that the introduction of the two-layer attention mechanism in the CNN-LSTM model improves the classification performance on Fer2013 and CK + data sets. Comparison with other neural networks that incorporate attention mechanism shows that our work has certain advantages.

## 2. Related Works

A convolution operator across the spatial domain is added to the network by Yang et al. to extract spatial features to automatically learn spectral spatial features from hyperspectral images [12]. Then, a bidirectional convolutional long and short-term memory network was built using a bidirectional LSTM to fully capture the spectral information. Nagaraju et al. proposed a fully learning-based method for pixel-level segmentation and classification of scene images-2D LSTM network, which achieves good performance on SIFT stream datasets [13], considering the complex spatial dependencies of natural scene image labels.

The model proposed by Lin et al. is composed of a deep LSTM network [14], eight encoders, and eight decoder layers. The attention mechanism is used to connect the bottom layer of the decoder to the top layer of the encoder, which improves parallelism, reduces the training time, and provides a new solution for neural machine translation. Fu et al. found that not all joints can provide information for action analysis in their study of 3D human action recognition [15]. Instead, irrelevant joints usually produce some noise. Attention mechanisms can be employed to focus on the joints that can provide information. Thus, a global context-aware attention LSTM network is proposed to achieve the aim of focusing on the most informative joints in each frame of the skeleton sequence, which shows excellent performance on 3D action recognition datasets.

During recent years, researchers have been trying to optimize the network model using the attention mechanism. For facial expression recognition tasks, Fernandez et al. present a new model, that is end-to-end network architecture with an attention [16]. Li et al. proposed a CNN with attention mechanism (ACNN) [17]. The ACNN can identify the occlusion regions of the facial expression image and focus on the most discriminative regions that are unoccluded.

## 3. Methodology

In this chapter, the specific composition of the two network architectures we designed is described. For the first one, a layer of attention mechanism is added to the CNN-LSTM network. For the other, a two-layer attention mechanism is introduced.

### 3.1. CNN-LSTM Facial Expression Recognition Network Model Incorporating an Attention Mechanism

**Figure 1:**
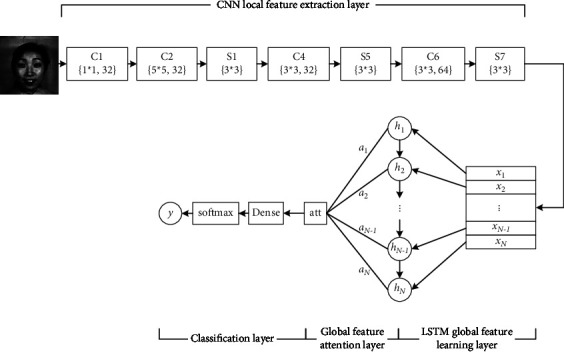
CNN-LSTM model the structure of global feature fusing attention mechanism. In CNN local feature extraction layer, *C* means the convolutional layer and *S* means the pooling layer. The elements in {} represent the size and the number of the convolution kernels, respectively. The convolutional layers and the pooling layers have step size of 1 and 2, respectively.

When the RNN is employed for facial expression classification tasks, the results of average sampling according to time steps are used as classification features. However, the average sampling method blurs the focus and cannot expand the advantages of valuable information. The attention mechanism can make the network focus on effective information, so it is employed to selectively process the information input to the classifier to optimize the classification performance of the neural network. The CNN-LSTM facial expression recognition network model incorporating an attention mechanism (CNN-ALSTM) proposed in this section mainly includes four parts: CNN local feature extraction layer, LSTM feature learning layer, global feature attention layer, and classification layer. The CNN-ALSTM model structure diagram is shown in Figure 1.

#### 3.1.1. CNN Local Feature Extraction Layer

This layer is mainly responsible for extracting the local abstract features of the facial expression image and serializing the generated feature vector as the input feature of the LSTM network. The CNN local feature extraction layer is composed of a 7-layer convolutional neural network, including 4 convolutional layers and 3 down-sampling layers. The parameters of each layer are shown in Figure 1.

Different from the employing the large convolution kernel in the original CNN model directly, the convolution layer C1 executes point-by-point convolution of the input data using a 1 × 1 convolution kernel. This is beneficial to increase the nonlinear representation of the input and improve the feature representation ability. In addition, the 1 × 1 point convolution kernel has few parameters, which can effectively reduce the network calculation complexity.

The pooling layer in the CNN local feature extraction layer uses the maximum pooling method to extract the strongest features. This can reduce the resolution of the feature map and reduce the computational complexity.

#### 3.1.2. LSTM Global Feature Learning Layer

The input sequence is *x*={*x*_1_, *x*_2_,…, *x*_*N*_}, and the input data at moment *n* is *x*_*n*_. The main work of this layer is roughly divided into four parts. First, the facial expression abstract feature vectors obtained by the CNN feature extraction layer are serialized as the input of the LSTM network. Then, according to the order of the time series, they are sequentially input into the main structure of the recurrent network. At the same time, the LSTM memory unit generates state information combining the current input data and historical information at each moment. Finally, the state information is passed to the next layer feature, which contains historical information at all previous moments.

#### 3.1.3. Global Feature Attention Layer

After the image is extracted by the LSTM network, the hidden state sequence of the LSTM network is defined as *h*={*h*_1_, *h*_2_,…, *h*_*N*_}, *h*_*n*_ is the hidden state at moment *n*. To select features that are more relevant to the classification task, a query vector q related to the facial expression recognition task is introduced. A scoring function is used to calculate the correlation between the hidden state and the query vector, which means that the higher the correlation between the hidden state and the classification task, the higher the score. For each hidden state, the correlation score can be obtained by the following equation:

(1) $$\begin{array}{c} s\left(h_{n},q\right)=h_{n}^{T}q\text{.} \end{array}$$

*s*(*h*_*n*_, *q*) is the attention scoring function.

After scoring the relevance of all hidden state tasks, the score is normalized to obtain the attention distribution *a*_*n*_ of the hidden state:

(2) $$\begin{array}{c} a_{n}=soft\;\max\;\left(s\left(h_{n},q\right)\right)=\frac{\exp\;\left(s\left(h_{n},q\right)\right)}{\overset{N}{\underset{j=1}{\sum }}\exp\left(s\left(h_{j},q\right)\right)}\text{.} \end{array}$$

The attention distribution *a*_*n*_ is the probability representation of the importance of the hidden state *h*_*n*_ in the facial expression recognition task.

The weighted average method is employed to fuse the information of all hidden sequences of the LSTM model, focusing on the most relevant features of the task. The following formula is the weighted feature representation of the sequence of hidden states:

(3) $$\begin{array}{c} att\left(h,q\right)=\overset{N}{\underset{n=1}{\sum }}a_{n}h_{n}\text{.} \end{array}$$

#### 3.1.4. Classification Layer

Finally, there is the classification layer. The classification layer is responsible for classifying the weighted features fused by the global feature attention layer.

### 3.2. CNN-LSTM Facial Expression Recognition Network Model Incorporating a Two-Layer Attention Mechanism

**Figure 2:**
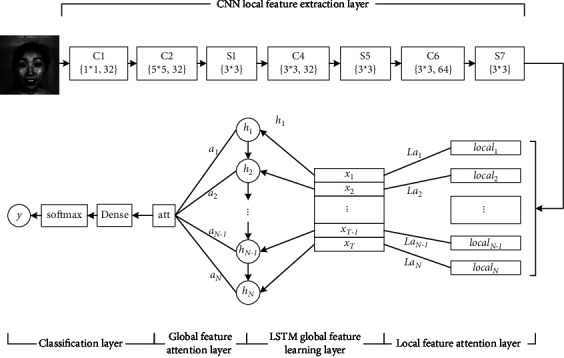
ACNN-ALSTM model the structure of local feature and global feature fusing attention mechanism.

An ACNN-ALSTM network model is proposed in this section. The network mines the local features through CNN [18, 19] and then uses the long-term memory capability of RNN to establish the spatial global dependence of the local features. LSTM is employed to compensate for the network instability when RNN processes long sequences. For the recognition of facial expression features, the proposed model further incorporates the attention mechanism based on the previous section, the process of which is similar to the CNN- ALSTM. The attention distribution is calculated for the hidden state of the LSTM, and then a weighted summation is performed on the hidden state. Afterwards, it is sent to the classifier as an optimized facial expression feature. The proposed model mainly includes five parts: CNN local feature extraction layer, local feature attention layer, LSTM feature learning layer, global feature attention layer, and classification layer. The ACNN-ALSTM model structure diagram is shown in Figure 2.

#### 3.2.1. CNN Local Feature Extraction Layer

The structure of this layer is exactly the same as the CNN local feature extraction layer in the previous section, so it is not repeated.

#### 3.2.2. Local Feature Attention Layer

The role of this layer is to strengthen the expression ability of essential local features. The query vector *q* related to facial expression recognition tasks is introduced. Thus, similar to the previous section, the correlation between each local feature *local*_*n*_ and the query vector can be calculated through the scoring function. Local features with high scores are more essential:

(4) $$\begin{array}{c} s\left({local}_{n},q\right)={local}_{n}^{T}q\text{.} \end{array}$$

*S*(*local*_*n*_, *q*) is the attention scoring function. After completing the task relevance scoring, the score is normalized to obtain the attention distribution *La*_*n*_ of the local features as shown in the following formula:

(5) $$\begin{array}{c} La_{n}=soft\;\max\;\left(s\left({local}_{n},q\right)\right)\text{.} \end{array}$$

The attention distribution *La*_*n*_ is the probability representation of the essential of the local feature *local*_*n*_. Then, weight each feature as the input of LSTM:

(6) $$\begin{array}{c} x_{n}=La_{n}∙{local}_{n}\text{.} \end{array}$$

*x*_*n*_ is the weighted local feature.

#### 3.2.3. LSTM Global Feature Learning Layer

The process at this layer is similar to that described in the previous section. The LSTM algorithm employs gating units and strong memory ability to establish the global spatial dependence of facial information.

#### 3.2.4. Global Feature Attention Layer

The hidden state output by LSTM is employed as the input of the global feature attention layer. The query vector q of the attention layer is defined as a learnable weight vector whose dimension is consistent with the number of hidden layer nodes of the LSTM network.

This layer implements the weighted fusion of the output features of the LSTM using the attention mechanism in two steps. First, the attention distribution *a*={*a*_1_, *a*_2_,…, *a*_*N*_} for the hidden state *h*={*h*_1_, *h*_2_,…, *h*_*N*_} of the LSTM network is calculated. Second, the weighted average result *att* of the hidden state is calculated according to the attention distribution.

The attention mechanism can increase the weight of the features that are most relevant to facial expression recognition tasks and achieve its dominant role in the feature fusion process, thereby improving the classification performance.

#### 3.2.5. Classification Layer

The classification layer is responsible for classifying the weighted features fused by the global feature attention layer. First, to reduce the dimensionality of the feature to the number of categories, the full connection is used for classification, and a 7-dimensional vector *e*^*V*^ is obtained. The symbol V corresponds to the attention scoring function *s*(*h*_*n*_, *q*) above. Then, a normalized formula (7) is formed combining with a softmax function. Using the softmax function, the facial expression features that are weighted and fused through the attention mechanism are classified. *S*_*i*_ represents the probability that the input sample belongs to the *i* th facial expression, *i* ∈ {1,2,…, 7}.

(7) $$\begin{array}{c} S_{i}=\frac{e^{V_{i}}}{\overset{7}{\underset{j=0}{\sum }}e^{V_{j}}}\text{.} \end{array}$$

Finally, the loss of the entire model is calculated by cross entropy, and the calculation method is shown as follows:

(8) $$\begin{array}{c} L\left(y,S\right)=-ylog\left(S\right)\text{.} \end{array}$$

*y* refers to the probability distribution in the real category, and *S* represents the probability distribution of the categories predicted by the model in this section.

## 4. Experiments

The data sets, simulations implementation, training details, and expression recognition results are described carefully in this part. As for the analysis of the experimental results, the effect of LSTM parameters on the facial expression features extracted by the model is experimentally studied. Besides, the performance of the methods proposed is confirmed by the expression recognition result.

### 4.1. Datasets

Two data sets are employed, namely, CK+ and Fer2013 data sets. Both data sets contain facial images of 7 emotions and they will be described in detail.

#### 4.1.1. Fer2013 Data Set [20]

The Fer2013 data set is mainly from the well-known data science competition platform Kaggle. The data is obtained by searching for image keywords through the Google search engine, including 35,887 gray-scale images with a resolution of 48 ∗ 48 pixels.  Table 1 shows the details of various expressions in the Fer2013 data set. The data set is rich and diverse since the images are crawled from the network, but the data contains a lot of noise, which is a challenge for facial expression recognition classification algorithms.

#### 4.1.2. CK + Data Set

The CK+ (Extended Cohn Kanade) data set was established in 2010 by the Patrick Lucey team and the Zara Ambadar team [21]. The database is a close-up of faces taken by 123 subjects according to certain expression requirements. There are 593 facial image data in the database, 327 of which include seven kinds of facial emotions. Since there is less data with expression tags in this data set, data enhancement processing is performed on 327 images with emotions [22]. First, face recognition is performed on the image to trim the invalid background. Then, all images are converted to 48 × 48 resolution. Furthermore, the images are flipped and rotated to increase the amount of data to about 3 times the original. At the same time, the brightness and saturation of the images are adjusted. The data distribution of the processed CK  + data set is shown in Table 2.

### 4.2. Implementation and Training Details

Two kinds of image classification experiments are designed to verify the effects of the two deep learning network models proposed in the paper on facial expression recognition. These experiments are implemented on the Linux16.04 64 bit operating system, using python3.6 and the Google framework tensorflow1.12.0-gpu for programming and implementation, and calling Open CV for data enhancement. NVIDIA Ge Force GTX1080ti graphics card with 16G video memory is employed. The FER2013 and CK + data sets are divided into training set, validation set, and test set according to the ratio of 8 : 2. The iteration is 5000 and print the training loss and error every 100 rounds. The loss and error of the test are printed every 400 rounds. Face images of the input model are rescaled to 48 × 48 pixels. The learning rate of the model is 0.001, and Adam optimizer and softmax regression are employed for calculation and classification [23, 24]. Cross entropy is used to calculate the loss function, and the batch size is 30.

It is found that the feature abstraction degree of the LSTM model of the image is related to the number of nodes in the hidden layer and the depth of the hidden layer. Therefore, the influence of the number of nodes in the hidden layer and the depth of the hidden layer on the effect of facial expression recognition is shown in Table 3.

### 4.3. Expression Recognition Results

We conducted two types of experiments. First of all, the experiment studied the influence of LSTM parameters on facial expression features extracted by the model. Then, a comparison about various variants of ACNN-ALSTM and related work is executed.

#### 4.3.1. The Influence of LSTM Model Parameters on Facial Expression Recognition

**Figure 3:**
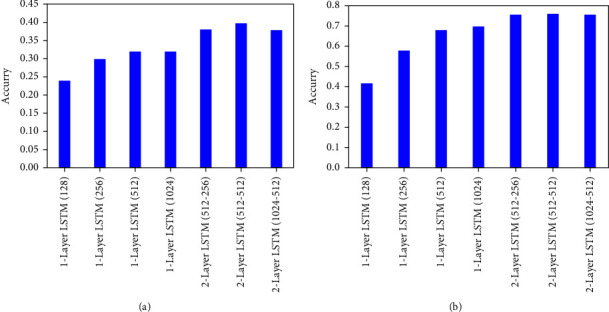
Influence of LSTM model parameters on two data sets: (a) Fer2013 data set; (b) CK + data set.

The paper considers two essential factors that affect the feature extraction of LSTM: the number of network layers and the number of hidden layer nodes. The classification results of differently constructed LSTM networks on the Fer2013 and the processed CK + data sets are shown in Figure 3.

According to the experimental results, the classification accuracy rate continues to increase as the number of nodes increases. At the same time, the abstract features learned from the image data of the LSTM network are more discriminative. However, the increase in the number of hidden layer nodes has an upper limit on the effect of increasing the recognition rate. From Figure 3, it can be found that the effect is the same when the hidden layer nodes are set to 512 and 1024. Besides, increasing the number of network layers can improve the facial expression recognition effect of the model. When the LSTM model is a two-layer network and the number of hidden layer nodes is 512-512, the classification accuracy of the model on the Fer2013 and CK  + datasets is relatively high.

#### 4.3.2. Comparison of Facial Expression Recognition Results of ACNN-ALSTM

**Figure 4:**
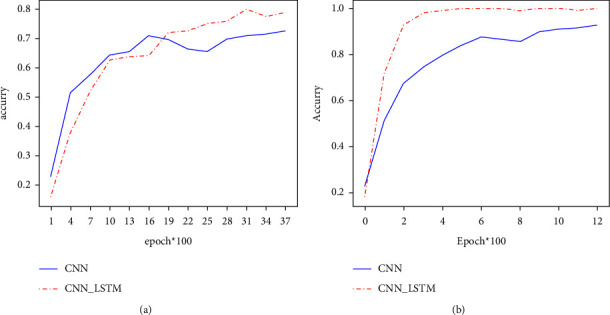
Comparison of the training results of the CNN-LSTM and the CNN model on two data sets: (a) Fer2013 data set; (b) CK  + data set.

CNN and CNN-LSTM models. The CNN-LSTM hybrid neural network is proposed by combining the advantages of CNN and LSTM to mine image features. The CNN and the CNN-LSTM models are demonstrated in Figures 4(a) and 4(b), and the two figures show the classification accuracy on Fer2013 and the processed CK  + dataset after every 100 trainings, respectively.

On the Fer2013 training set, the classification accuracy of the CNN-LSTM hybrid model surpassed the CNN model after 1900 iterations. On the CK + training set, the training classification accuracy of the CNN-LSTM hybrid model is always better than the CNN model. It can be seen that the CNN-LSTM hybrid model can effectively recognize facial expressions.

CNN-LSTM model and its various variants. These models are trained on the Fer2013 and the processed CK + training sets.  Table 4 shows the average test results obtained through multiple experiments on the test set. In the table, ACNN-LSTM represents that the local feature is fused with the attention mechanism. The CNN-ALSTM represents that the global feature is fused with the attention mechanism. The ACNN-ALSTM model represents that the local feature and the global feature are both fused with the attention mechanism.

Compared with that only the local feature or that only global feature is fused with the attention mechanism, the effect of both local feature and global feature are fused with the attention mechanism is better. At the same time, it can be found that the effect of fusing the attention mechanism in the global feature is better than that in the local feature. The above-given research results show that adding an attention mechanism to the CNN-LSTM model can highlight essential features and improve the classification effect.

ACNN-ALSTM and related work. Although some results cannot be directly compared due to different experimental setups and different preprocessing methods, it is demonstrated in Table 5 that the proposed method can yield a feasible and promising recognition rate for static facial images.

## 5. Discussion

It is obvious that the efficiency and accuracy of recognition can be promoted by making sufficient use of key parts of facial expression. However, the utilization of the key parts of the existing algorithm can be further improved. Thus, the CNN-ALSTM and the ACNN-ALSTM incorporating an attention mechanism are proposed in this paper. Simulation results indicate that the proposed algorithm can further improve the recognition rate compared with other researchers' work, although both their algorithms and ours incorporating attention mechanisms. At the same time, this work demonstrates the effectiveness of attentional mechanisms for exploiting key information about facial expressions.

## 6. Conclusion

In this work, a novel ACNN-ALSTM model for facial expression recognition that incorporates a two-layer attention mechanism is proposed. Comparative experimental results indicate that the introduction of the two-layer attention mechanism improves the classification performance of the system more significantly than that of the CNN-LSTM model and its variants. We also investigate the effect of the different network layers and the number of nodes in the hidden layers of the LSTM model on expression recognition. The results indicate that appropriately increasing the network depth and the number of hidden layer nodes is beneficial to improve the recognition accuracy of the LSTM model. In future work, we want to train the network for extreme conditions such as dark, light, and occlusion [25, 26].

## Figures and Tables

**Table 1:** Fer2013 data set.

Expression	Total	Anger	Disgust	Fear	Glee	Dump	Surprise	Neutral
Tag	—	0	1	2	3	4	5	6
Quantity	35887	4953	547	5121	8989	6077	4002	6198

**Table 2:** Data distribution after data enhancement for CK+ (extended Cohn Kanade) data set.

Expression	Total	Anger	Disgust	Fear	Glee	Dump	Surprise	Despise
Quantity	981	135	177	75	207	84	249	54

**Table 3:** LSTM model parameters settings.

Number of hidden layers	Number of hidden layer nodes (corresponds to each layer)
**1**	128
**1**	256
**1**	512
**1**	1024
**2**	512, 256
**2**	512, 512
**2**	1024, 512

**Table 4:** Comparison of classification accuracy of four models.

Method	Fer2013 (%)	CK+ (%)
CNN-LSTM	74.1	95.3
ACNN-LSTM	75.5	97.7
CNN-ALSTM	77.4	99.1
ACNN-ALSTM	**78.2**	**99.7**

**Table 5:** Comparison of classification accuracy with related work.

Method	Fer2013 (%)	CK+ (%)
pACNN [17]	74.3	97.0
FERAtt [16]	74.8	97.5
ACNN-ALSTM	**78.2**	**99.7**

## Data Availability

https://www.kaggle.com/c/challenges-in-representation-learning-facial-expression-recognition-challenge/data.
